# Does induction of labor without a medical indication explain the overall increase in the induction rate: an observational study before and after the ARRIVE trial

**DOI:** 10.1186/s12884-025-07403-8

**Published:** 2025-03-25

**Authors:** Isabelle Attali, Julie Cormier, François Goffinet, Camille Le Ray

**Affiliations:** 1 Port Royal Maternity Unit, Assistance Publique-Hôpitaux de Paris, Hôpital Cochin, FHU préma, 123 Bd de Port-Royal, Paris, 75014 France; 2 Centre of Research In Epidemiology and Statistics, Obstetrical, Perinatal and Paediatric Epidemiology EPOPé Research Team, Université Paris Cité, IHM Santé des femmes, INSERM, INRAE, Paris, U1153 France

**Keywords:** Induction of labor, Delivery, Caesarean section rate, Epidemiology

## Abstract

**Background:**

The rate of induction of labor increased particularly after the publication of ARRIVE trial conducted in low-risk primiparous patients without medical indication. However, this increase of induction rate does not seem to concern this population alone. Our aim was to understand how induction rate have evolved according to its indications and the impact on cesarean rate.

**Methods:**

This was a retrospective observational study in a tertiary university maternity unit, including all women who gave birth between January 1st 2014 and December 31th 2021, at more than 24 weeks of gestation with a liveborn infant weighing ≥ 500 g (*N* = 9,523). We described the frequency of induction and caesarean section per year within the maternity unit. We differentiated two study periods: 2014–2017 and 2018–2021. We used the Grenoble classification to analyse the contribution of each of group to the overall induction rate and calculated the absolute and relative difference in induction rate for each group between the two periods. We analysed changes in the risk of caesarean section in each of the groups.

**Results:**

The overall induction rate increased from 19.3 to 27.4% between 2014 and 2021 (*p* < 0.01). The cesarean section rate for women who underwent induction decreased significantly from 29.5% in 2014 to 25.2% in 2021 (*p* < 0.01). The induction rate moderately increased in the group corresponding to induction of labor “without medical indication” (relative difference of 14.9%; 95%CI [6.0;21.0]). The groups with the greatest increase in their induction rate between the two study periods were the breech group (relative difference of 66.7% 95%CI [49.0;83.0]) and the fetal pathology induction group (relative difference of 75.5% 95%CI [61.2;90.1]). The rate of cesarean among inducted women reduced significantly in the group of “multiple pregnancies” (aOR = 0.6; 95%CI [0.4;0.9]) and in the group of “maternal pathologies” (aOR = 0.8; 95%CI [0.6;0.9]). For the group 8 “induction without medical induction” the reduction was not significant (aOR = 0.8; 95%CI [0.8;1.2]).

**Conclusion:**

From 2014 to 2021, we observed a marked increase in the induction rate in our maternity unit. This increase was not associated with a change of the cesarean rate. Induction of labor without medical indication represent only a small part of the induction rate.

**Supplementary Information:**

The online version contains supplementary material available at 10.1186/s12884-025-07403-8.

## Background

Induction of labor is an increasingly common obstetric procedure worldwilde, affecting 25.8% of patients in France according to the latest National Perinatal Survey (*Enquête Nationale Périnatale (ENP*)) conducted in 2021 [1] whereas the rate was 22.0% in 2016 and stable between 2010 and 2016 according to the same survey. The procedure has also increased worldwide [2] and it is probably partly attributable to the results of the ARRIVE trial (A Randomized Trial of Induction Versus Expectant Management) published by Grobman and al. in 2018 [3]. The ARRIVE trial reported in low-risk nulliparous women that induction of labor at 39 weeks’ gestation (WG) does not increase neonatal morbidity compared with expectant management and is associated with a reduced risk of caesarean section. These results have been confirmed by subsequent studies, some of which even suggest a clear reduction in perinatal mortality [2, 4]. With regard to this reduction in the risk of emergency caesarean section, some meta-analyses support these results in favor of induction of labor [4–6], while others show no association [7–10].

Our question was therefore to evaluate whether this increase in induction rates occurred only for low-risk primiparous women or for the general population of women and whether it concerned only inductions without medical indication or whether other indications were affected. The Grenoble classification [11] was specifically designed to study the indications for induction of labor, and thus makes it possible to identify groups of induction of labor without any medical indication. This tool validated by a Delphi method corresponds to a simple, robust classification of 8 groups clinically sound, simple and clear.

Our primary objective was to compare the induction rate according to the Grenoble classification between two study periods (2014–2017 and 2018–2021). Our secondary objective was to compare the caesarean section rate among induced patients according to the Grenoble classification between these two periods.

## Methods

We conducted an observational retrospective study in a tertiary university public maternity unit performing around 5200 deliveries annually between January 1st 2014 and December 31th 2021. In our unit, physicians based their decision of induction on local protocols and all patient files are discussed by the staff in the morning. The labor induction technique was chosen based on the Bishop score: if ≤ 3, Dinoprostone Vaginal Delivery System (PROPESS) was used; for scores between 4 and 5, Dinoprostone gel (PROSTINE E2) was administered; and for scores ≥ 6, amniotomy and oxytocin were applied.

**Fig. 1:**
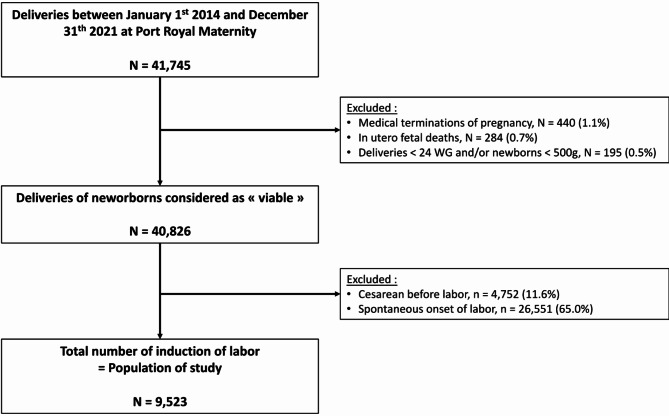
Flow chart

We included all women who gave birth during the study period (*n* = 41,745) (Fig. 1). We excluded in utero fetal deaths (IUFD *n* = 284), medical terminations of pregnancy (*n* = 440), deliveries before 24 weeks’ gestation and/or infants weighing less than 500 g (*N* = 195), patients who had a caesarean section before labor (*N* = 4,752) and those who had a spontaneous labor (*N* = 26,551).

All data were collected from the hospital computer database.

We used the Grenoble classification [11] (Supplementary Fig. 1) to separate the women into 8 groups according to the indication for induction of labor:

- Group 1: Multiple pregnancies,
- Group 2: Single breech pregnancy,
- Group 3: Preterm single cephalic pregnancy (term less than 37 weeks’ gestation),
- Group 4: Single cephalic pregnancy with prelabor rupture of membranes at term (37 weeks’ gestation and above),
- Group 5: Single cephalic pregnancy, late term and post term (41weeks’ gestation and over), intact membranes,
- Group 6: Single cephalic pregnancy, term between 37 and 40 weeks, 6days with maternal pathology indicating induction, intact membranes,
- Group 7: Single cephalic pregnancy, term between 37 and 40weeks, 6days with fetal pathology indicating induction, intact membranes,
- Group 8: Single cephalic pregnancy, term between 37 and 40weeks, 6days, induced without medical indication, intact membranes.

We first described and compared number of deliveries, caesarean section rate, induction rate and caesarean section rate among patients induced by year within the maternity unit, between 2014 and 2021 by a chi square test.

We then described maternal characteristics (age, BMI, geographical origin, history of hypertension or diabetes, parity) and obstetric characteristics by year (type of pregnancy (outcome of human assisted reproduction or not), gestational age at delivery, mode of delivery and birthweight.

We then compared two study periods: 2014–2017 and 2018–2021. We chose 2018 as a threshold year following the publication of the ARRIVE study [3]. 

We calculated for each of the Grenoble group its contribution to the overall induction rate (as a percentage that is the effective of women with an induction rate in the group divided by the total population of women induced).

We compared the overall induction rate for the two periods studied and calculated the absolute difference (rate of the 2nd period– rate of the 1st period) and the relative difference ((rate of the 2nd period– rate of the 1st period) / rate of the 1st period) in induction rate for each group between the two periods. For each calculation, we have added 95% confidence intervals (CI) and p value for the absolute difference.

Finally, we compared the caesarean section rate among the induced women in each of the 8 groups between the two periods. We performed a univariate and then a multivariate analysis using a logistic regression model with adjustment for parity and geographical origin in order to estimate changes in the risk of caesarean section between the two periods for each group in the Grenoble classification.

The data were presented as follows: continuous variables are written as means +/- SD and categorical variables as numbers: n (%).

Continuous variables were compared by a Student’s t test and categorical variables by a Chi2 test. A p-value < 0.05 was considered significant. In univariate and multivariate analysis we estimated crude odds ratios and adjusted odds ratios with 95% confidence intervals (CI). Analyses were performed using Jamovi software version 2.4.0.

The study was conducted in accordance with the Declaration of Helsinki. Under French regulations, this study is exempt from IRB review because it is an observational study using anonymized data from medical records. Women are informed that their records can be used for the evaluation of medical practices and are provided the option to opt out of these studies (agreement of National Data Protection Authority (*Commission Nationale de l’Informatique et des Libertés*,* CNIL n°1*,*755*,*849*)). Human Ethics and Consent to Participate declarations are not applicable for this study in France.

## Results

Between 2014 and 2021, 9523 patients were induced in the maternity unit. We observed a significant overall increase in the induction rate from 19.3% in 2014 to 27.4% in 2021 (*p* < 0.01) (Table 1). During the study period, the caesarean section rate for all deliveries in our maternity unit remained stable. Among induced patients, the caesarean section rate decreased significantly between 29.5% in 2014 to 25.2% in 2021 (*p* < 0.01) (Table 1).

**Table 1:** Induction of labor and caesarean section rates during the study period

	TOTAL		2014		2015		2016		2017		2018		2019		2020		2021	
	N	%	N	%	N	%	N	%	N	%	N	%	N	%	N	%	N	%
**Total of deliveries**	40,826		5,135		4,929		5,163		5,200		5,178		5,188		4,907		5,126	
Total of caesarean sections	10,004	24.5	1,358	26.4	1,176	23.9	1,312	25.4	1258	24.2	1243	24.0	1261	24.3	1155	23.5	1,241	24.2
**Total of induction of labor**	9,523	23.3	993	19.3	1,086	22.0	1,099	21.3	1135	21.8	1254	24.2	1314	25.3	1238	25.2	1,404	27.4
**Caesarean sections among inductions**	2,462	25.9	293	29.5	275	25.3	313	28.5	319	28.1	309	24.6	292	22.2	307	24.8	354	25.2

**Table 2:** Population characteristics during the study period

	TOTAL		2014		2015		2016		2017		2018		2019		2020		2021	
	*N*	%	*N*	%	*N*	%	*N*	%	*N*	%	*N*	%	*N*	%	*N*	%	*N*	%
Total of induction of labor	9523	23.3	993	19.3	1086	22.0	1099	21.3	1135	21.8	1254	24.2	1314	25.3	1238	25.2	1404	27.4
**Maternal age (y)**	33	+/- 7.3	32.8	+/- 6.9	33,2	+/- 6.9	32,7	+/- 7.0	32,8	+/- 7.0	33,2	+/- 6.9	33.3	+/- 6.8	33.3	+/- 6.9	32.9	+/- 6.8
**Prepregnancy BMI**	23,8	+/- 7.5	24	+/- 5.2	23,7	+/- 5.2	23,6	+/- 5.2	24,3	+/- 5.1	23,6	+/- 5.0	23.5	+/- 5.0	24,0	+/- 5.1	23.8	+/- 5.0
**Country of birth**																		
Metropolitan France	4766	50	462	46.5	510	47.0	535	48.7	534	47.0	617	49.2	683	52.0	635	51.3	790	56.3
Other	4757	50	531	53.4	576	53.1	564	51.3	601	52.9	637	50.8	631	48.1	603	48.7	614	43.8
**Hypertension**	174	1.8	11	1.1	18	1.7	25	2.3	26	2.3	28	2.2	24	1.8	22	1.8	20	1.4
**Diabetes**	234	2.5	22	2.2	26	2.4	25	2.3	30	2.6	33	2.6	34	2.6	36	2.9	28	2.0
**Primiparous**	5664	59.5	604	60.8	666	61.3	697	63.4	686	60.4	762	60.8	767	58.4	703	56.8	779	55.5
**Pregnancy obtained by HAR**	1127	11.8	98	9.9	134	12.3	109	9.9	134	11.8	181	14.4	163	12.4	154	12.4	154	11.0
**Gestational age at delivery**	39.4	+/- 1.9	39.5	+/-1.9	39,5	+/ 1.7	39,3	+/ 1.9	39.4	+/ 1.9	39.3	+/ 1.9	39,3	+/ 1.9	39.2	+/ 1.9	39.4	+/ 1.9
**Mode of delivery**																		
Spontaneous vaginal delivery	6175	64,8	590	59,4	710	65,4	677	61,6	729	64,2	820	65,4	910	69,3	813	65,7	926	66,0
Instrumental vaginal delivery	886	9,3	110	11,1	101	9,3	109	9,9	87	7,7	125	10,0	112	8,5	118	9,5	124	8,8
Caesarean section	2462	25,9	293	29,5	275	25,3	313	28,5	319	28,1	309	24,6	292	22,2	307	24,8	354	25,2
**Birthweight (g)**	3263	+/-574	3274	+/- 551	3288	+/-549	3259	+/- 550	3270	+/- 550	3239	+/- 545	3237	+/- 542	3237	+/- 546	3299	+/- 539

All continuous variables (maternal age, prepregnancy BMI, gestational age at delivery, birthweight) are expressed as mean +/- SD

BMI: body mass index; HAR: human assisted reproduction; SD: standard deviation; y: years

Table 2 described the women and obstetrics characteristics by year over the study period. Women were most often from metropolitan France and multiparous over the time.

**Table 3:** Evolution of the indications of induction rates according to the Grenoble classification

Group of the Grenoble classification	2014–2017		2018–2021		ABSOLUTE DIFFERENCE	95% CI	*P* value	RELATIVE DIFFERENCE	95% CI
	*N*	%	*N*	%	%			%	
**Group 1**: Multiple pregnancies	281	1.4	318	1.6	+ 0.2	[0.1 ; 0.3]	0.03	+ 13.0	[7.3 ; 19.2]
**Group 2**: Single breech pregnancy	36	0.2	62	0.3	+ 0.1	[0.1 ; 0.2]	0.01	+ 66.7	[49.0 ; 83.0]
**Group 3**: Preterm single cephalic pregnancy (term less than 37 weeks gestation)	241	1.2	280	1.4	+ 0.2	[0.1 ; 0.3]	0.01	+ 16.1	[8.0 ; 28.0]
**Group 4**: Single cephalic pregnancy with prelabor rupture of membranes at term (37 weeks gestation and above)	862	4.2	1087	5.3	+ 1.11	[0.9 ; 1.3]	< 0.01	+ 26.3	[9.0 ; 43.0]
**Group 5**: Single cephalic pregnancy, late term and post term (41 weeks gestation and over), intact membranes	1196	5.9	1329	6.5	+ 0.7	[0.5 ; 0.8]	< 0.01	+ 11.4	[5.0 ; 13.2]
**Group 6**: Single cephalic pregnancy, term between 37 and 40 weeks, 6 days with maternal pathology indicating induction, intact membranes	909	4.5	1039	5.1	+ 0.7	[0.4 ; 0.9]	< 0.01	+ 14.6	[10.3 ; 21.0]
**Group 7**: Single cephalic pregnancy, term between 37 and 40weeks, 6days with fetal pathology indicating induction, intact membranes	319	1.6	560	2.8	+ 1.2	[0.9 ; 1.4]	< 0.01	+ 75.5	[61.2 ; 90.1]
**Group 8**: Single cephalic pregnancy, term between 37 and 40 weeks, 6 days, induced without medical indication, intact membranes	274	1.3	314	1.5	+ 0.2	[0.1 ; 0.3]	< 0.01	+ 14.9	[6.0 ; 21.0]
**Total of induction rates**	4313	21.1	5210	25.5	+ 4.4	[4.0 ; 4.8]	< 0.01	+ 20.9	[15.5 ; 25.0]

The percentages are calculated by dividing the effectives of each group by the total of deliveries in a category: *n* = 20 427 for the 2014–2017 category and *n* = 20 399 for the 2018–2021 category

When comparing the two study periods, 2014–2017 and 2018–2021, the overall induction rate increased from 21.1 to 25.5% (*p* < 0.01) (Table 3). The absolute difference between each group shows the contribution of each to the overall increase in the induction rate. All groups contributed to the total increase in the induction rate.

Within each group, we quantified the increase in induction rate by their relative difference. The groups that increased their induction rate the most between the two periods were the breech group (group 2) (relative difference of 66.7% 95%CI [49.0;83.0]) and the fetal pathology induction group (group 7) (relative difference of 75.5% 95%CI [61.2;90.1]). For all the other groups, the increase was moderate but significant (relative difference between 13.0% and 26.3%), including the group corresponding to induction of labor without medical indication (group 8) (relative difference of 14.9% 95%CI [6.0;21.0]).The rate of missing or unclassifiable data using Grenoble classification was 4.5% for period 1 and 4.2% for period 2.

Overall, between the two periods, the caesarean section rate among induced patients decreased significantly (27.8% vs. 24.2%; aOR = 0.9; 95% CI [0.8; 0.9]). This decrease remained significant after adjustment for geographical origin and parity (aOR of 0.9; 95% CI [0.8; 0.9]) (Table 4). This reduction was particularly significant in group 1 “multiple pregnancies” (40.9% vs. 28.9%; aOR = 0.6; 95% CI [0.4; 0.9]) and in group 6 “maternal pathologies” (30.1% vs. 23.0%; aOR = 0.8; 95% CI [0.6; 0.9]). For the group 8 “induction without medical induction” the reduction was not significant (14.2% vs. 11.1%; aOR = 0.8; 95% CI [0.8; 1.2]).

**Table 4:** Risk of caesarean section among women induced according to the Grenoble classification

Risk of caesarean section	2014–2017	2018–2021	Unadjusted OR	95% CI	aOR*	95% CI
**Group 1**: Multiple pregnancies	40.9	28.9	0.6	[0.4; 0.8]	0.6	[0.4; 0,9]
**Group 2**: Single breech pregnancy	63.9	48.4	0.5	[0.2; 1.2]	0.5	[0,2; 1,4]
**Group 3**: Preterm single cephalic pregnancy (term less than 37 WG)	34.0	35.7	1.1	[0.8; 1.5]	0.9	[0,6; 1.3]
**Group 4**: Single cephalic pregnancy with prelabor rupture of membranes at term (37 WG and above)	22.4	21.2	0.9	[0.7; 1.2]	1.0	[0,8; 1,2]
**Group 5**: Single cephalic pregnancy, late term and post term (41 WG and over), intact membranes	28.0	27.3	1.0	[0.8; 1.2]	1.0	[0.9; 1,2]
**Group 6**: Single cephalic pregnancy, term between 37 and 40 weeks, 6 days with maternal pathology indicating induction, intact membranes	30.1	23.0	0.7	[0.6; 0.8]	0.8	[0,6; 0,9]
**Group 7**: Single cephalic pregnancy, term between 37 and 40 weeks, 6 days with fetal pathology indicating induction, intact membranes	32.0	25.9	0.7	[0.5; 1.0]	0.8	[0,6; 1,1]
**Group 8**: Single cephalic pregnancy, term between 37 and 40 weeks, 6 days, induced without medical indication, intact membranes	14.2	11.1	0.8	[0.5; 1.2]	0.8	[0.5; 1,4]
**TOTAL**	27.8	24.2	0.8	[0.8; 0.9]	0.9	[0,8; 0,9]

CI: confidence interval; WG: weeks’ gestation; OR: odds ratio; aOR: adjusted odds ratio; Rates expressed as percentages

*Adjustment for parity (primiparous or not) and geographical origin (Metropolitan France or not)

## Discussion

In our maternity unit, there was an increase in the overall induction rate, rising from 19.3 to 27.4% between 2014 and 2021. We can observe that this increase is linked to a general increase in all groups of the Grenoble classification and not to a significant increase in the rate of induction in the group of women without a medical indication. The increase in the rate of induction was greater for breech and fetal pathologies.

The caesarean section rate for all deliveries remained stable but among induced patients, the caesarean section rate decreased significantly between 29.5% in 2014 and 25.2% in 2021.

The first strengths of our study is its originality and its large sample. In addition, it is based on a recent classification composed of 8 groups considered to be clinically relevant and mutually exclusive which enabled us to identify a group of induction without medical indication. Lastly, this study was carried out in a maternity hospital where practices are homogeneous and decisions on induction are taken by staff and protocols rather than on an individual basis.

However, there are limits, the retrospective design of our study did not allow a certain effective of women to be classified (4.5% of patients for the 1st period and 4.2% for the 2nd period) due to missing data. Moreover, the single-centre design of the study limits the external validity of the results. We have a lot more high-risk patients, so the distribution of groups in the classification is not representative of all French hospitals, but only of level three university hospitals. In addition, the Grenoble classification does not allow us to study the indication of induced labor. Women are classified according to the type of pregnancy, foetal presentation and term of delivery, but not according to the exact reason for induction, which limits the interpretability of the results. However, the use of a classification specifically developed to assess induction of labor allows the analyses to be reproduced in other maternity units and possibly compared with our own.

The increase in the rate of induction, particularly for the breech (group 2) and foetal pathology (group 7) groups, can be linked to changes in maternity protocols according to recent publications concerning breech delivery and macrosomia. Firstly, the study by Gaillard et al. [12] assessed severe neonatal morbidity and mortality in patients who had undergone induction for a breech fetus, compared with those who had undergone a scheduled caesarean section. There was no significant difference in neonatal morbidity and mortality between the two groups. The results have led to changes in practice in our maternity unit, where cervix ripening in patients with a breech fetus is now an option. Secondly, the DAME randomised controlled trial, which compared induction and expectant management among women with a suspected “large for gestational age” (LGA). The results of this study showed a significant reduction in the rate of neonatal morbidity, leading to a systematic proposition of induction for women in this clinical situation [13].

The absence of an increase in the caesarean section rate is consistent with certain data in the current literature published on the subject [3, 13–16]. On the other hand, the increase in induction rate was greater from 2018, which supported our hypothesis of 2018 as a year of change in induction practices.

Results showed a significant decrease in the rate of caesarean sections among women who underwent induction of labor during the study period. This decline was particularly notable among women induced for twin pregnancies (group 1), although no certain explanation for this trend was identified. A similar reduction was observed in cases involving maternal pathologies (group 6). One possible explanation is that the criteria for labor induction have expanded, leading to an increase in inductions among women with moderate pathologies or symptoms that could previously have led to a debate between induction and expectant management. This association may also suggest that induction of labor may be performed in women at lower risk of caesarean section.

Furthermore, in our maternity unit, we have not observed any increase in inductions without medical indication. Indeed following the ARRIVE trial publication, we have taken the decision not to offer systematic induction for convenience, pending the results of other studies and before changing our practices. However, in certain cases, we have authorized some inductions without medical indication, mainly in multiparous women with a favorable cervix according to the French recommendations published in 2005 [17]. Thus, we are expecting the results of French ARRIVE (NCT04799912) [18], trial based on the promising results of ARRIVE trial to test the hypothesis that elective induction of labor at 39 weeks of gestation in low risk nulliparas leads to a lower cesarean delivery rate than expectant management.

## Conclusion

An increase in the induction rate was observed in our maternity unit between 2014 and 2021. All induction groups are affected by this increase but breech and fetal pathology are the two indications for induction that have increased the most during the study period. Using the Grenoble classification, we have identified that induced labor without medical indication remained stable and represented only a small proportion of induced labor in our maternity unit. The results of ARRIVE trial did not have an impact solely on the population targeted by the study i.e. low risk nulliparous women. Global cesarean rate remained stable during the study period but decrease among induced women.

## Electronic supplementary material

Below is the link to the electronic supplementary material.

Supplementary Material 1

## Data Availability

The anonymized datasets used and analyzed during the study are available from the corresponding author on reasonable request.

## Abbreviations

aOR: Adjusted odds ratio
ARRIVE: A Randomized Trial of Induction Versus Expectant Management
CI: Confidence interval
LGA: Large for gestational age
LGA: Large for gestational age
WG: Weeks’ gestation
