# Supplementary material for: Does induction of labor without a medical indication explain the overall increase in the induction rate: an observational study before and after the ARRIVE trial
Source: BMC Pregnancy Childbirth. 2025 Mar 25;25:349. doi: 10.1186/s12884-025-07403-8 (PMC11934459; doi:10.1186/s12884-025-07403-8)
Supplement: Supplementary file 1 — Supplementary Material 1 [file 12884_2025_7403_MOESM1_ESM.docx]

**APPENDIX**


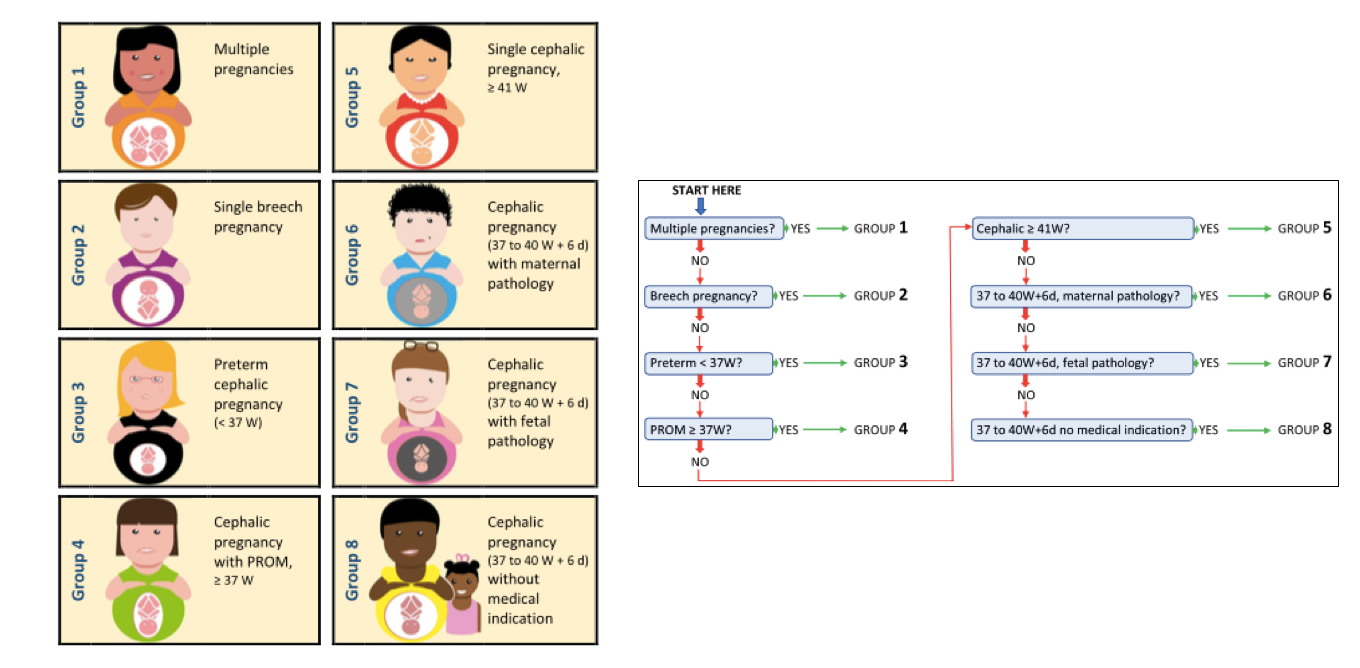


*Source : Vanneaux et al. ; BMC Pregnancy and Childbirth (2022) 22:143*

**Supplementary Figure 1:** The Grenoble classification of artificial induction of labor into 8 groups and the system to classify each patient in one of the groups of Grenoble classification
